# Training, Anthropometric, and Physiological Characteristics in Men Recreational Marathon Runners: The Role of Sport Experience

**DOI:** 10.3389/fphys.2021.666201

**Published:** 2021-04-12

**Authors:** Pantelis T. Nikolaidis, Vicente Javier Clemente-Suárez, Daniela Chlíbková, Beat Knechtle

**Affiliations:** ^1^Exercise Physiology Laboratory, Nikaia, Greece; ^2^School of Health and Caring Sciences, University of West Attica, Athens, Greece; ^3^Faculty of Sports Sciences, Universidad Europea de Madrid, Madrid, Spain; ^4^Grupo de Investigación en Cultura, Educación y Sociedad, Universidad de la Costa, Barranquilla, Colombia; ^5^Centre of Sports Activities, Brno University of Technology, Brno, Czechia; ^6^Institute of Primary Care, University of Zurich, Zurich, Switzerland

**Keywords:** body fat, endurance training, maximal oxygen uptake, skinfold thickness, sport history, training volume

## Abstract

The aim of the present study was to examine the physiological and training characteristics in marathon runners with different sport experiences (defined as the number of finishes in marathon races). The anthropometry and physiological characteristics of men recreational endurance runners with three or less finishes in marathon races (novice group, NOV; *n* = 69, age 43.5 ± 8.0 years) and four or more finishes (experienced group, EXP; *n* = 66, 45.2 ± 9.4 years) were compared. EXP had faster personal best marathon time (3:44 ± 0:36 vs. 4:20 ± 0:44 h:min, *p* < 0.001, respectively); lower flexibility (15.9 ± 9.3 vs. 19.3 ± 15.9 cm, *p* = 0.022), abdominal (20.6 ± 7.9 vs. 23.8 ± 9.0 mm, *p* = 0.030) and iliac crest skinfold thickness (16.7 ± 6.7 vs. 19.9 ± 7.9 mm, *p* = 0.013), and body fat assessed by bioimpedance analysis (13.0 ± 4.4 vs. 14.6 ± 4.7%, *p* = 0.047); more weekly training days (4.6 ± 1.4 vs. 4.1 ± 1.0 days, *p* = 0.038); and longer weekly running distance (58.8 ± 24.0 vs. 47.2 ± 16.1 km, *p* = 0.001) than NOV. The findings indicated that long-term marathon training might induce adaptations in endurance performance, body composition, and flexibility.

## Introduction

During the last decades, an increase of recreational marathon runners and annual races conducted all over the world has been observed (Knechtle et al., 2018; Vitti et al., 2020). Accordingly, an increased scientific interest has been focused on the evaluation of the physiological characteristics of these runners (Salinero et al., 2017; Nikolaidis and Knechtle, 2018). In addition, the role of health-related physical fitness components (e.g., body composition, aerobic capacity, flexibility, and muscle strength) for human’s health and well-being has been well-established (Lopez-Torres et al., 2019; McCormack et al., 2020). Previous studies showed that low body fat percentage (BF) was a key success factor in ultra-endurance races (Barandun et al., 2012), fact related with the large metabolic requirement (Clemente-Suarez, 2015) that could compromise even protein status, increasing the protein catabolism and muscle breakdown (Jamart et al., 2012). In this line, other researchers highlighted the importance of other parameters for this eliciting sport events founding how training schedule (Clemente-Suarez and Nikolaidis, 2017), odontological and nutritional variables (Belinchon-deMiguel et al., 2019), as well as emotional and personality constructs (Lane and Wilson, 2011). Considering the popularity of this sport (Vitti et al., 2020), it would be of great interest to examine long-term adaptations of health-related physical fitness to regular training in recreational marathon runners.

To investigate the effect of endurance training in recreational marathon runners, two methodological approaches might be applied relying on either longitudinal (Iwasaki et al., 2003) or cross-sectional study design (Mosher et al., 2010; Bishop et al., 2019; Lee, 2019). For the purpose of the present study, sport experience–reflecting long-term endurance training–was defined as the number of finished marathon races. So far, the relationship of the number of finished endurance races with training and physiological characteristics has been not well studied, and previous research provided conflicting findings (Knechtle et al., 2010b; Salinero et al., 2017). For instance, it has been observed that the number of finished marathons did not correlate with marathon race time nor differ among recreational runners with different race times (Salinero et al., 2017). On the other hand, in 100 km ultra-marathon runners, race time correlated with the number of finished 100 km ultra-marathons, i.e., the larger the number of finisher races, the fastest the race time (Knechtle et al., 2010b).

Although physiological and training characteristics have been well studied in recreational marathon runners especially with regard to performance level, less information has been available about the variation of these characteristics by sport experience (Salinero et al., 2017). Examining these characteristics in recreational marathon runners of different sport experiences would be a novel approach with practical applications. The aim of the present study was to examine the physiological and training characteristics in marathon runners with different sport experiences. For the purpose of the study, “sport experience” referred to the number of finished marathons. Although it was acknowledged that other indices of sport experience existed (e.g., training years), the number of finished marathons might be considered a practical cut-off especially in the case of recreational marathon runners who were the focus of this study. It was hypothesized that groups of men recreational marathon runners of different sport experiences would exhibit similar training and physiological characteristics, considering recent findings (Salinero et al., 2017).

## Materials and Methods

This study has been part of a larger project on the physiological and psychological aspects of marathon runners, and detailed description of the study design and experimental procedures might be accessed elsewhere (Nikolaidis and Knechtle, 2018). Briefly, 135 recreational marathon runners, who finished the “Athens Authentic Marathon” in 2017, participated in the present study and provided written informed consent. All procedures were in accordance with the Declaration of Helsinki, and the local Institutional Review Board provided approval (EPL 2017/7). For the purpose of the present study, the anthropometric and physiological characteristics of men recreational endurance runners with three or less finishes in marathon races (novice group, NOV; *n* = 69, age 43.5 ± 8.0 years; 2.0 ± 0.8 finishes; 4.1 ± 2.2 years of regular running training; personal best marathon running time 4:20 ± 0:44 h:min) and four or more finishes (experienced group, EXP; *n* = 66, 45.2 ± 9.4 years; 9.4 ± 7.3 finishes; 9.7 ± 7.0 years of regular running training; personal best time 3:44 ± 0:36 h:min) were compared. NOV had median 2 finishes (interquartile range, IQR 1–3 finishes) and 4 years of training (IQR 3–5 years), whereas EXP had median 6 finishes (IQR 5–12 finishes) and 7 years of training (IQR 6–12.5 years). The study design was cross-sectional, where all data were collected during a single testing session.

Information on the number of finished marathon races, personal best marathon time (h:min), and number of weekly training days and weekly running distance (km) was recorded in a paper and pencil questionnaire. Body weight and height were measured using a scale (HD-351; Tanita, Arlington Heights, IL, United States) and stadiometer (SECA Leicester, United Kingdom), respectively. Body mass index was calculated as the ratio of body weight to height squared (kg m^–2^). Body composition (BF) was tested using bioimpedance analysis (BIA; Tanita BC-545, Arlington Heights, IL, United States) and skinfold thickness (Harpenden, West Sussex, United Kingdom) at 10 sites according to Parizkova’s method (Eston and Reilly, 2009). The inter- and intra-rater reliability of skinfold thickness measurements was 0.99 for the researcher who administered this assessment. Flexibility was evaluated through a sit-and-reach test (Mayorga-Vega et al., 2014), and muscle strength through squat jump (SJ) and countermovement jump (CMJ) (Microgate Engineering, Bolzano, Italy) (Aragon-Vargas, 2000). For both flexibility and jump tests, two trials were performed with 1-min break between trials and tests, and the best score was recorded for further analyses. A graded exercise test (GXT) on a treadmill using inclination + 1% evaluated VO_2_max using a gas analyzer (Fitmate Pro, Cosmed, Rome, Italy). Fitmate Pro was an automated metabolic analyzer relying on a representative small sample of expired volume in a dynamic mixing chamber (Nieman et al., 2007). In the GXT, a modified Conconi protocol was performed, where running speed increased by 1 km/h every minute with an initial speed set at 8 km/h (Conconi et al., 1982; Chrismas et al., 2017).

IBM SPSS v.20.0 (SPSS, Chicago, IL, United States) and GraphPad Prism v.7.0 (GraphPad Software, San Diego, United States) were used for the statistical analyses. Descriptive statistics (mean and standard deviation) were calculated for all data. The level of significance was set at α = 0.05. Independent *t* test examined the differences between EXP and NOV. Pearson correlation coefficient r examined the relationship of the number of finishes in marathon races with training, anthropometric, and physiological variables.

## Results

**FIGURE 1:**
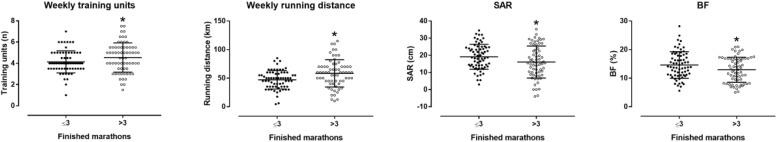
Weekly training units and running distance, sit-and-reach test, and body fat percentage by number of marathon finishes. Error bars represented standard deviations. *p < 0.05. SAR, sit-and-reach test; BF, body fat percentage assessed by bioimpedance analysis.

**FIGURE 2:**
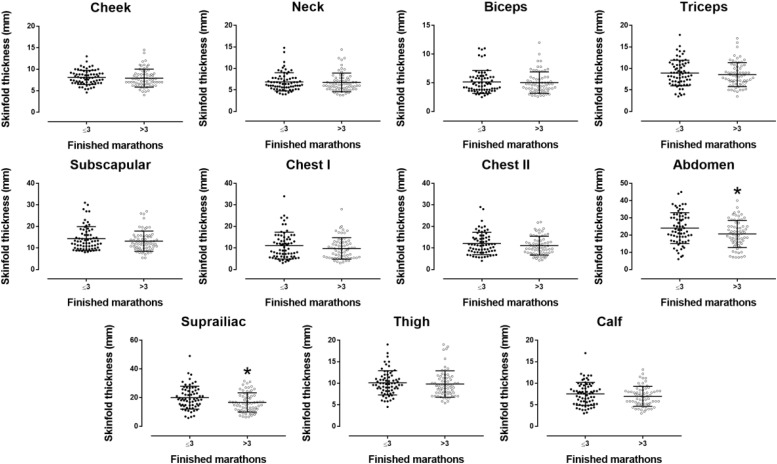
Skinfold thickness of 11 anatomical sites by number of marathon finishes. Error bars represented standard deviations. *p < 0.05.

EXP had faster personal best marathon time than NOV (3:44 ± 0:36 vs. 4:20 ± 0:44 h:min, *p* < 0.001, respectively) (Table 1). Furthermore, they had lower flexibility (15.9 ± 9.3 vs. 19.3 ± 15.9 cm, *p* = 0.022), abdominal (20.6 ± 7.9 vs. 23.8 ± 9.0 mm, *p* = 0.030) and iliac crest skinfold thickness (16.7 ± 6.7 vs. 19.9 ± 7.9 mm, *p* = 0.013), and body fat assessed by BIA (13.0 ± 4.4 vs. 14.6 ± 4.7%, *p* = 0.047); more weekly training days (4.6 ± 1.4 vs. 4.1 ± 1.0 days, *p* = 0.038); and longer weekly running distance (58.8 ± 24.0 vs. 47.2 ± 16.1 km, *p* = 0.001) than NOV (Figures 1, 2). The number of finishes in marathon races correlated with SJ (*r* = –0.41, *p* = 0.021) and CMJ (*r* = –0.38, *p* = 0.032), weekly training days (*r* = 0.19, *p* = 0.030), running distance (*r* = 0.25, *p* = 0.004), and years of running training (*r* = 0.63, *p* < 0.001).

**TABLE 1:** Comparison between experienced (EXP) and novice (NOV) recreational marathon runners.

**Variable**	**NOV (*n* = 69)**	**EXP (*n* = 66)**
Number of finishes	2.0 ± 0.8	9.4 ± 7.3^‡^
History of running training (years)	4.1 ± 2.2	9.7 ± 7.0
Best time (h:min)	4:20 ± 0:44	3:44 ± 0:36^‡^
Weekly training days	4.1 ± 1.0	4.6 ± 1.4*
Weekly distance (km)	47.2 ± 16.1	58.8 ± 24.0^†^
Age (years)	43.5 ± 8.0	45.2 ± 9.4
Height (cm)	176 ± 5	177 ± 7
Body weight (kg)	77.5 ± 9.1	76.4 ± 9.6
BMI (kg m^–2^)	25.1 ± 2.7	24.4 ± 2.5
BF_*skinfolds*_ (%)	18.2 ± 4.0	17.2 ± 4.1
BF_*BIA*_ (%)	14.6 ± 4.7	13.0 ± 4.4
SAR (cm)	19.3 ± 7.3	15.8 ± 9.3*
VO_2_max (ml min ^–1^ kg^–1^)	47.4 ± 8.2	49.3 ± 7.9
SJ (cm)	24.8 ± 4.4	23.9 ± 4.2
CMJ (cm)	26.6 ± 4.8	25.1 ± 4.7
Cheek (mm)	8.1 ± 1.6	8.0 ± 2.1
Chin (mm)	6.8 ± 2.1	6.8 ± 2.2
Triceps (mm)	8.9 ± 3.0	8.5 ± 2.8
Subscapular (mm)	14.1 ± 5.8	13.1 ± 4.7
Pectoral (mm)	11.0 ± 6.2	9.8 ± 5.0
Chest II (mm)	12.0 ± 5.2	11.0 ± 4.4
Abdomen (mm)	23.8 ± 9.0	20.6 ± 7.9*
Iliac crest (mm)	19.9 ± 7.9	16.7 ± 6.7*
Patella (mm)	10.1 ± 2.8	9.9 ± 3.1
Proximal calf (mm)	7.5 ± 2.6	7.0 ± 2.3

Data are presented as mean ± standard deviation. BMI, body mass index; BF, body fat percentage; VO_2_max, maximal oxygen uptake; SJ, squat jump; CMJ, countermovement jump. *p < 0.05, ^†^*p* < 0.01, and ^‡^*p* < 0.001.

## Discussion

The main findings of the present study were that more experienced runners had (a) faster personal best marathon time, (b) lower flexibility, (c) lower abdominal and iliac crest skinfold thickness, (d) lower fat assessed by BIA, and (e) more weekly training days and longer weekly running distance than their less experienced counterparts. Furthermore, the number of finishes in marathon races correlated negatively with SJ and CMJ and positively with weekly training days and running distance.

EXP was faster than NOV by 36 min, which was partially in agreement with previous studies (Till et al., 2016; Gordon et al., 2017). It was previously observed that fast marathon runners had more sport experience and weekly training volume than slow runners (Gordon et al., 2017), which was also in consonance with longer distance and running events as ultra-endurance mountain races (Belinchon-Demiguel and Clemente-Suarez, 2019). On the other hand, a study of the Australian marathons did not find any relationship between marathon race time and years of training; however, this result should be considered with caution since the sample size was relatively small (*n* = 19) (Till et al., 2016). It should be reported that, despite the abovementioned 36 min difference in race time, EXP and NOV did not differ in VO_2_max, which was considered as a main determinant of marathon race time (Gordon et al., 2017; Nikolaidis and Knechtle, 2018). An explanation of this observation was that performance might rely on other factors, such as higher maximal lactate steady state, closer anaerobic threshold to the VO_2_max (Fontana et al., 2009), as well as non-physiological factors (e.g., motivation) in addition to physiology (Waskiewicz et al., 2019). In this context, it was not surprising that different physiological profiles can lead to the same level of performance as shown in a recent case study (Louis et al., 2020). In addition to the number of finished marathon races, EXP had also more years of running training than NOV, and the overall larger sport background of EXP might account for other performance-related adaptations to marathon training, such as the ability to optimally distribute their effort during a race. Several recent works (Aschmann et al., 2018; Nikolaidis et al., 2019; Hernando et al., 2020) have shown the importance of maintaining a relatively even pace from the start line to the finish line of a marathon. This ability might be developed with increasing expertise and could be a determining factor for performance in long distance races.

The lower flexibility in EXP than in NOV might be attributed to potential differences in running economy and suggested an adaptation of musculotendinous structures to long-term training. Although running economy was not measured in participants, it would be reasonable to assume that EXP being the fastest group might run more metabolically economically. In turn, high running economy has been shown to relate with small flexibility (Jones, 2002; Trehearn and Buresh, 2009), indicating stiffer musculotendinous structures (Drew et al., 2011). A muscular structure with greater rigidity, which logically does not imply a structural complication that increases the fragility and risk of injury, will have a greater reactivity that will allow a lower loss of elastic energy in each stride, taking advantage of a more efficient way mechanical energy is stored in the musculotendinous structure (Nelson et al., 2001). Accordingly, the negative correlation of the number of finished marathons with SJ and CMJ might reflect a training adaptation to endurance training. This finding was in agreement with previous research (Martinez-Navarro et al., 2020) that observed a trend that fast runners exhibited relatively low SJ, too.

With regard to the variation of skinfold thickness and body fat between the two groups, EXP had smaller thickness in two skinfolds (abdominal and iliac crest) and lower BF estimated by BIA than NOV. These differences might be attributed to adaptations of body composition to long-term endurance training. It has been observed that the sum of skinfolds was moderately and positively related to marathon race time (Hagan et al., 1981; Salinero et al., 2017), i.e., fast runners had lower BF than slow runners. The increased workload of non-active tissue in the running movement was described as a limitation in almost all sport in which repeated movement is necessary, especially in long distance event (Ramos-Campo et al., 2014; Belinchon-deMiguel and Clemente-Suarez, 2018). In addition, the higher BF of NOV combining with the similar weight of both groups highlighted the lower fat-free mass of NOV. This observation could also explain the lower performance of NOV not having so much muscle mass that may be involved in muscle contraction, and finally in the production of force in each stride (Suárez et al., 2011).

In addition, differences were found in training habits, where EXP had more weekly training days and running distance than NOV. This finding might be attributed to the faster race time of EXP compared with NOV. It has been shown that the weekly training units and distance were largely and negatively related to marathon race time (Hagan et al., 1981), i.e., the more the weekly training units and the longer the distance covered, the faster the race time. Another study reported moderate and negative relationship of weekly training distance with marathon race time (Salinero et al., 2017). In contrast, other studies have shown how training velocity correlated with the ultra-endurance race time (Knechtle et al., 2010a), also training with lower volume and an incremental distribution of aerobic workload obtain higher improvements in aerobic performance, basic for endurance races (Clemente Suarez and Gonzalez-Rave, 2014; Clemente-Suarez et al., 2017, 2018a). Other factors, such as the intensity and periodization of training workloads, could also affect performance (Clemente-Suarez et al., 2015, 2018b); therefore, it would be interesting to analyze these parameters coupled with the volume of training for future investigations.

A limitation of our study was the use of a particular cut-off to define EXP and NOV based on the number of finishes in marathon races. It was acknowledged that sport experience might be also estimated by other methods, e.g., time since starting regular endurance training. Nevertheless, it was considered that this variable would be hard to estimate due to difficulties of participants to recall it; e.g., often, the participants reported periods with no engagement in regular training. Thus, the number of finishes was considered as a more “quantifiable” estimate of sport experience, and caution would be needed to generalize the findings to other estimates of sport experience. On the other hand, strength was the inclusion of a wide range of training, anthropometric, and physiological variables providing insights on this topic. The information about the differences in training, anthropometric, and physiological variables by experience level was a novel finding with practical applications. Coaches and fitness trainers working with recreational marathon runners might apply these findings to evaluate the physical readiness of their athletes and monitor the effectiveness of their training program.

## Conclusion

The findings indicated that long-term marathon training might induce adaptations in endurance performance, body composition, and flexibility. An interpretation of the lower score of flexibility in the more experienced group might be its relationship with running economy. The negative relationship of the number of finishes with indices of muscle strength (jump tests) suggested a negative adaptation of muscle strength to endurance training.

## Data Availability Statement

The raw data supporting the conclusions of this article will be made available by the authors, without undue reservation.

## Ethics Statement

The studies involving human participants were reviewed and approved by This study has been part of a larger project on physiological and psychological aspects of marathon runners, and detailed description of the study design and experimental procedures might be accessed elsewhere (Nikolaidis and Knechtle, 2018). Briefly, 135 recreational marathon runners, who finished the “Athens Authentic Marathon” in 2017, participated in the present study and provided written informed consent. All procedures were in accordance with the Declaration of Helsinki and the local Institutional Review Board provided approval. The patients/participants provided their written informed consent to participate in this study.

## Author Contributions

PN collected all the data, performed the analyses, and drafted the first manuscript. VC-S, DC, and BK helped in finishing the manuscript. All authors contributed to the article and approved the submitted version.

## Conflict of Interest

The authors declare that the research was conducted in the absence of any commercial or financial relationships that could be construed as a potential conflict of interest.
